# Inflammatory infiltration into placentas of *Neospora caninum* challenged cattle correlates with clinical outcome of pregnancy

**DOI:** 10.1186/1297-9716-45-11

**Published:** 2014-01-31

**Authors:** Germán J Cantón, Frank Katzer, Stephen W Maley, Paul M Bartley, Julio Benavides-Silván, Javier Palarea-Albaladejo, Yvonne Pang, Sionagh H Smith, Mara S Rocchi, David Buxton, Elisabeth A Innes, Francesca Chianini

**Affiliations:** 1 Moredun Research Institute, Pentlands Science Park, Bush Loan, Penicuik EH26 0PZ, UK; 2 Instituto Nacional de Tecnología Agropecuaria (INTA), EEA Balcarce, Balcarce 7620, Argentina; 3 Instituto de Ganadería de Montaña (CSIC-ULE), León 24346, Spain; 4 Biomathematics and Statistics Scotland, Edinburgh EH9 3JZ, UK; 5 Royal (Dick) School of Veterinary Studies and Roslin Institute, University of Edinburgh, Edinburgh EH25 9RG, UK

## Abstract

Infection with *Neospora caninum* stimulates host cell-mediated immune responses, which may be responsible for placental damage leading to bovine abortion. The aim of this study was to compare immune responses in the bovine placenta, following experimental infection in different stages of pregnancy. Placentomes were examined by immunohistochemistry and inflammation in early gestation was generally moderate to severe, particularly in the placentas carrying non-viable foetuses, whereas it was milder in later stages, mainly characterised by the presence of CD3^+^, CD4^+^ and γδ T-cells. This distinctive cellular immune response may explain the milder clinical outcome observed when animals are infected in later gestation.

## Introduction, methods and results

Bovine abortion is one of the major constraints to the livestock industry and *Neospora caninum* is recognised as a major cause of reproductive loss in cattle [1]. The pathogenesis of bovine neosporosis is complex and only partially understood, and the reasons why only some animals abort remain unclear [2]. In just a few days following experimental infection of pregnant dams, *N. caninum* can cause lethal lesions in brains and hearts of foetuses [3]. In addition, there is evidence that infection with *N. caninum* triggers a Th1-type immune response at the materno–foetal interface along with release of pro-inflammatory cytokines [4]. This type of immune response, if exacerbated in the placental tissues, could be detrimental to the pregnancy by initiating an inflammatory process which can damage the placenta and disrupt the vascular supply of nutrients, leading to abortion [5-7]. Therefore, in some cases, the foetus might be killed by the shift from a homeostatic maternal Th2-type towards a detrimental Th1-type immune response during gestation as has been demonstrated in mice [8].

The aim of this study is to compare the cellular infiltrate into the placental tissues in pregnant cattle experimentally inoculated with *N. caninum* in early (day 70), mid (day 140) and late gestation (day 210), in order to explain the different clinical outcomes previously reported [6,7,9]. A summary of these studies is available in Additional file 1.

Animals were sacrificed at 2, 4, 6 and 8 week intervals following experimental inoculation. Immediately after euthanasia, three, five and ten randomly selected placentomes were sampled from the animals inoculated respectively at day 70, 140 and 210 of gestation and fixed in zinc salts fixative (ZSF) (pH 7.0-7.4). After three days of fixation, tissues were paraffin wax-embedded. The phenotype of the cells present in the inflammatory infiltrate following inoculation with *N. caninum* in early [10], mid and late [11] gestation was carried out, using immunohistochemistry (IHC) with monoclonal antibodies (mAbs) recognizing different bovine immune cell subsets (see Additional file 2). All the IHC slides were assessed using a previously reported scoring methodology [11,12].

**Figure 1:**
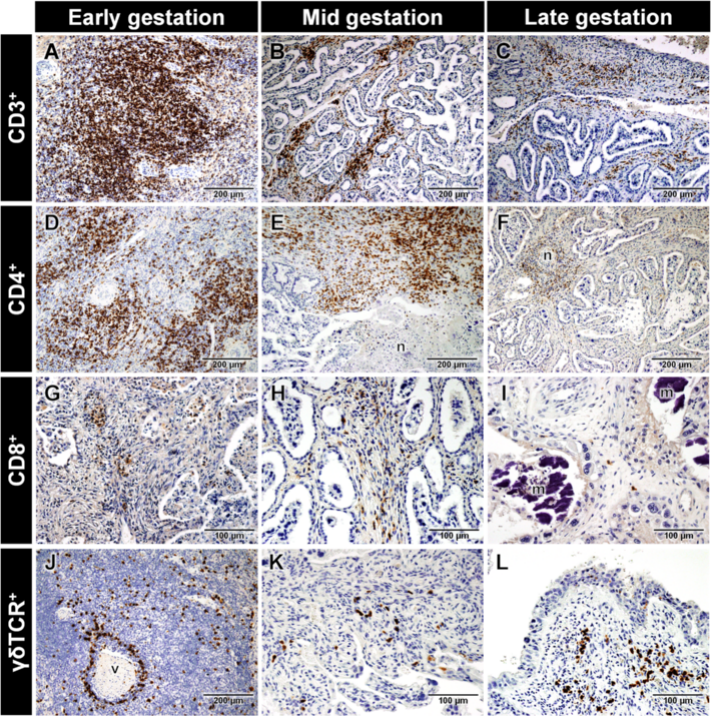
**Lymphocyte infiltration in placentomes from *N. caninum*-inoculated dams in early, mid and late gestation. (A)** Large area of infiltration of CD3^+^ cells in the placentome of a dam carrying a non-viable foetus in early gestation. **(B)** Moderate infiltration of CD3^+^ cells in the maternal caruncle stalk of the placentome of a dam in mid gestation. **(C)** Mild infiltration of CD3^+^ cells in the maternal caruncle of a dam in late gestation. **(D)** Severe infiltration of CD4^+^ cells in the placentome of a dam carrying a non-viable foetus in early gestation. **(E)** Severe infiltration of CD4^+^ cells in caruncle surrounding a large area of necrosis (n) in the placentome of a dam in mid gestation. **(F)** Mild infiltration of CD4^+^ cells in the caruncle surrounding necrotic foetal villi (n) in the placentome of a dam in late gestation. **(G)** Mild infiltration of CD8^+^ cells in the maternal caruncle of a dam carrying a non-viable foetus in early gestation. **(H)** Mild infiltration of CD8^+^ cells in the maternal caruncle of a dam in mid gestation. **(I)** Rare CD8^+^ cells infiltrating the maternal caruncle surrounding areas of mineralization (m), in a dam in late gestation. **(J)** Moderate infiltration of γδTCR^+^ cells surrounding a blood vessel (v) in the maternal caruncle of a dam carrying a non-viable foetus in early gestation. **(K)** Mild infiltration of γδTCR^+^ cells in the maternal caruncle of a dam in mid gestation. **(L)** Mild γδTCR^+^ cells infiltrating the maternal caruncle in a dam in late gestation. Counterstained with haematoxylin.

The individual scores from randomly sampled placentomes were used to calculate a single mean score for each animal, similar to previous descriptions [11]. Given the limited group sample sizes, culling time effects were assumed to be non-significant and pooled data were used in all experiments. For each cell type, Mann–Whitney tests were used: (1) to assess differences in scores between *N. caninum*-inoculated (from now on termed “challenged”) and negative control (from now on termed “control”) dams, and (2) to compare scores between dams carrying a dead foetus or having an empty uterus in the time of *post mortem* examination (from now on termed “non-viable”) and those carrying a live foetus (from now on termed “viable”). Overall comparisons of scores between gestational stages were conducted by Kruskal-Wallis tests, followed by Mann–Whitney tests (FDR-adjusted *p*-values) for pair-wise comparisons. A 5% significance level was considered for all statistical tests. The immune cell infiltration of placentomes from dams challenged at day 70 was already described by Maley et al. [10] and was reassessed and scored for the current work as reported previously [11]. IHC was performed and infiltration scores were assessed in placental samples collected at day 140 of gestation. Statistical analysis was carried out to compare the new data with those reported for 210 days of gestation [11]. Examples of CD3^+^, CD4^+^, CD8^+^ and γδ T lymphocyte infiltration in the placentomes from challenged dams in early, mid and late gestation are presented in Figure 1. Results of the comparison between early, mid and late gestation for each immune cell type are shown in Additional file 3. The mean and standard errors of the mean (SEM) of the different immune cell scores in early, mid and late gestation are shown in Tables 1 and 2. All animal procedures complied with the Animals (Scientific Procedures) Act 1986 and were approved by the Moredun Research Institute ethics committee.

**Table 1:** **Mean (± SEM) of immune cell infiltration scores on placentomes collected from *N. caninum*-inoculated (“challenged”) and negative control (“control”) dams in early, mid and late gestation**

**Cell type**	**Early gestation**		**Mid gestation**		**Late gestation**	
	**Control**	**Challenged (SC + IV)**	**Control**	**Challenged**	**Control**	**Challenged**
**n**	**8**	**16**	**3**	**6**	**4**	**11**
CD68^+^	1.79 (± 0.17)^*a*^	2.83 (± 0.16)^*b,A*^	0.87 (± 0.13)^*a*^	2.07 (± 0.20)^*aAB*^	1.25 (± 0.07)^*a*^	1.80 (± 0.08)^*aB*^
CD3^+^	1.15 (± 0.06)^*a*^	2.94 (± 0.12)^*bA*^	0.43 (± 0.12)^*a*^	2.12 (± 0.21)^*bAB*^	0.25 (± 0.07)^*a*^	0.87 (± 0.08)^*bB*^
CD4^+^	0.96 (± 0.09)^*a*^	2.49 (± 0.15)^*bA*^	0.37 (± 0.11)^*a*^	1.70 (± 0.25)^*aAB*^	0.15 (± 0.06)^*a*^	0.44 (± 0.06)^*aB*^
CD8^+^	0.46 (± 0.09)^*a*^	1.13 (± 0.07)^*bA*^	0.20 (± 0.11)^*a*^	0.77 (± 0.10)^*aAB*^	0.08 (± 0.04)^*a*^	0.20 (± 0.04)^*aB*^
γδTCR^+^	0.83 (± 0.07)^*a*^	1.39 (± 0.06)^*bA*^	0.17 (± 0.06)^*a*^	1.17 (± 0.08)^*bA*^	0.25 (± 0.07)^*a*^	0.63 (± 0.05)^*bB*^
NKp46^+^	0.83 (± 0.07)^*a*^	1.12 (± 0.06)^*bA*^	0.20 (± 0.10)^*a*^	0.87 (± 0.06)^*bAB*^	0.35 (± 0.09)^*a*^	0.88 (± 0.04)^*bB*^
CD79_αcy_^+^	0.96 (± 0.04)^*a*^	0.92 (± 0.05)^*aA*^	1.07 (± 0.07)^*a*^	1.20 (± 0.07)^*aB*^	0.96 (± 0.02)^*a*^	0.96 (± 0.02)^*aA*^

n: number of animals per group.

^*a,b*^Different lowercase superscript letters indicate statistically significantly different infiltration scores between control negative and *N. caninum*-inoculated dams (Mann–Whitney test) in each experiment (early, mid and late gestation) (*p* ≤ 0.05).

^*A,B*^Different uppercase superscript letters indicate statistically significantly different overall infiltration scores for each cell marker between *N. caninum*-inoculated dams in early, mid and late gestation (Kruskal-Wallis test) (*p* ≤ 0.05).

**Table 2:** **Mean (± SEM) of immune cell scores on placentomes collected in early gestation from dams inoculated with *N. caninum *** **carrying non-viable (dead calves or empty uterus) or viable foetuses (alive calves) at culling**

**Cell type**	**Non-viable**	**Viable**
CD68^+^	2.55 (± 0.25)^*a*^	3.09 (± 0.19)^*a*^
CD3^+^	3.43 (± 0.10)^*a*^	2.50 (± 0.17)^*b*^
CD4^+^	2.90 (± 0.21)^*a*^	2.11 (± 0.20)^*b*^
CD8^+^	1.40 (± 0.09)^*a*^	0.89 (± 0.07)^*b*^
γδTCR^+^	1.73 (± 0.06)^*a*^	1.09 (± 0.05)^*b*^
NKp46^+^	1.30 (± 0.08)^*a*^	0.95 (± 0.07)^*b*^
CD79_αcy_^+^	0.75 (± 0.07)^*a*^	1.07 (± 0.07)^*b*^

^*a,b*^Different letters indicate significantly different scores (*p* ≤ 0.05).

In early gestation the CD68^+^ cell scores were higher in placentomes from challenged animals than in controls (*p* = 0.016), although no differences were established between the placentas carrying viable and non-viable foetuses (*p* > 0.05). In mid gestation, no differences in CD68^+^ scores were detected between placentomes from challenged and control dams (*p* = 0.091). Overall differences were established for CD68^+^ scores between challenged dams in early, mid- and late pregnancy (*p* = 0.019); but when pair-wise compared, differences were only detected between early and late gestation (*p* = 0.015). Overall the scores from control dams in the three stages of gestation were not different.

In early gestation the CD3^+^ cell scores were higher in placentomes from challenged dams than in those from controls (*p* < 0.001) and in those from dams carrying non-viable foetuses than in those carrying viable foetuses (*p* = 0.008). In mid gestation, the CD3^+^ cell scores were higher in placentomes from challenged dams than in those from controls (*p* = 0.020). Overall inter-experiment differences in CD3^+^ scores from challenged animals were established (*p* < 0.001), and pair-wise comparison showed that CD3^+^ scores were higher in early (*p* < 0.001) and mid-gestation (*p* = 0.024) compared to late gestation. Overall differences were also found in CD3^+^ scores in the placentomes from control dams in the three stages of gestation (*p* = 0.003).

In early gestation the CD4^+^ scores were higher in the placentomes from challenged dams than in the controls (*p* < 0.001). CD4^+^ scores in challenged dams carrying non-viable foetuses were higher compared to the ones carrying viable foetuses (*p* = 0.043). In mid gestation, there were no CD4^+^ scores differences between challenged and controls (*p* > 0.05)*.* Overall inter-experiment scores were found to be different between placentomes from the challenged dams from the three stages of gestation (*p* < 0.001), although when pair-wise analysed, higher scores were only found in early gestation compared with the late gestation (*p* < 0.001). In the controls, overall CD4^+^ scores were also different in three stages of gestation (*p* = 0.005).

In early gestation higher CD8^+^ scores were found in the placentomes from challenged animals than in the controls (*p* = 0.003), and challenged dams carrying non-viable foetuses had higher CD8^+^ scores than those carrying viable foetuses (*p* = 0.006). No differences were observed in the CD8^+^ scores between challenged and control dams in mid gestation (*p* > 0.05). Overall CD8^+^ scores from challenged animals were different between the three stages of gestation (*p* < 0.001). When they were pair-wise analysed, scores were only higher in early (*p* < 0.001) and mid gestation (*p* = 0.016) when compared with late gestation. No overall differences were observed in the CD8^+^ T cell scores in the placentomes from control dams (*p* > 0.05).

In early gestation higher γδTCR^+^ scores were found in the placentomes from challenged cows than in the controls (*p* = 0.016), and these were higher in challenged dams carrying non-viable foetuses than in those carrying viable foetuses (*p* = 0.005). In mid gestation, higher γδTCR^+^ scores were found in the placentomes from challenged cows than in controls (*p* = 0.018). Overall inter-experiment scores were compared for the challenged animals and differences were established for γδTCR^+^ between the three stages of gestation (*p* < 0.001); when pair-wise compared, higher scores were only found in early (*p* < 0.001) and mid (*p* = 0.005) gestation compared with the late stage. Overall, inter-experiment γδTCR^+^ scores were different in the placentomes from control dams in the three stages of gestation (*p* = 0.004).

In early gestation NK scores were higher in the placentomes from challenged dams than in the controls (*p* = 0.002) and in the same period, higher NK scores were found in the challenged animals carrying non-viable foetuses than in those carrying viable foetuses (*p* = 0.001). In mid gestation, NK scores were also higher in the placentomes from challenged dams than in those from controls (*p* = 0.018). When overall NK scores were contrasted in challenged dams in the three stages of gestation, differences were found (*p* = 0.019); nevertheless, when they were pair-wise compared, higher scores were established between early and late gestation (*p* = 0.040). Overall differences were observed in the NK scores found in placentomes from control dams in the three stages of gestation (*p* = 0.004).

In early and mid gestation no differences were observed in the CD79_αcy_^+^ scores in placentomes from challenged and controls dams (*p* > 0.05). However, CD79_αcy_^+^ scores were lower in placentomes from challenged dams in early gestation carrying non-viable foetuses than in those carrying viable foetuses (*p* = 0.009). Overall there were differences in the CD79_αcy_^+^ score comparison between challenged animals in the three stages gestation (*p* = 0.019); however, when they were pair-wise contrasted, differences were only detected between early and mid gestation (*p* = 0.045), and between mid and late gestation (*p* = 0.023). No overall differences were observed in the CD79_αcy_^+^ scores in placentomes from control dams in the three stages of gestation (*p >* 0.05).

## Discussion

Previous studies have hypothesised that the placental cellular immune response may play a major role on the pathogenesis of bovine neosporosis [13,14]. However, very few studies have been carried out in different stages of gestation and they were usually generating qualitative data [10,13]. The present study produced semi-quantitative data allowing for the first time a comparison of the consequences of *N. caninum* infection at different stages of bovine gestation. However, we cannot rule out that the results obtained in this study were influenced by different breeds used in the three experiments [6,7,9] as differences in susceptibility to *Neospora* infection have been observed among livestock breeds in some surveys [15].

Using this new scoring methodology we were able to establish clear differences in the placental inflammatory infiltrate from animals infected with *Neospora* in different stages of gestation. Moreover, in early gestation these differences were evident in placentas carrying viable or non-viable foetuses. These data strongly support the hypothesis of immune-mediated pathogenicity of bovine neosporosis.

Higher macrophage scores were found in the *Neospora* infected animals in early gestation, when compared with later in gestation. This heavier infiltrate could trigger an intense adaptive immune response leading to injury of the maternal-placental junction and later endanger the foetus [16]. More studies are needed to further characterize this CD68^+^ cell population, and possibly differentiate the infiltration of M1 and M2 subtypes [17].

A positive association between several T lymphocyte (CD3, CD4, CD8 and γδ) subsets and occurrence of abortion was also established in the current study. These cells play a role in the development and maintenance of a Th1 biased response [16,18-21] that has been suggested to be dangerous to pregnancy and foetal survival [4,16,20].

The results presented in this paper, improve the understanding of the immunopathogenesis of bovine neosporosis. However, more studies aimed in the further characterisation of this immune response and evaluation of the role of pro-inflammatory cytokines is needed in order to extend the understanding of this disease.

## Competing interests

The authors declare that they have no competing interests.

## Authors’ contributions

FC, GC, EAI and FK conceived this study and participated in its design and coordination. DB, JB, FK, SWM, PMB, YP, MR, FC and EAI participated in the necropsy and sampling of the animals. GC, YP and SWM carried out the IHC analysis of the samples. GC has scored all the IHC slides. JPA performed the statistical analysis. GC, FC, SHS, and FK have written the manuscript; with inputs from all authors. All authors read and approved the final manuscript.

## Supplementary Material

Additional file 1 **Summary of findings of the previous experiments.** Clinical findings and transplacental infection results following experimental inoculation with *N. caninum* at early, mid and late gestation. Click here for file

Additional file 2 **mAb used to identify different immune cell phenotypes in the placentomes.** List of the mAb used to overnight incubate the placentomes during the IHC. Click here for file

Additional file 3 **Infiltration scores in placentomes from negative control and *N. caninum*-inoculated cows.** Mean infiltration score for placentomes collected from negative control (black bars) and *N. caninum* inoculated (grey bars) cows during the early, mid and late gestation experiments. Error bars indicate standard error of the means. Click here for file
