# Supplementary material for: Inflammatory infiltration into placentas of Neospora caninum challenged cattle correlates with clinical outcome of pregnancy
Source: Vet Res. 2014 Jan 31;45(1):11. doi: 10.1186/1297-9716-45-11 (PMC3922085; doi:10.1186/1297-9716-45-11)
Supplement: Additional file 1 — Summary of findings of the previous experiments. Clinical findings and transplacental infection results following experimental inoculation with N. caninum at early, mid and late gestation. [file 1297-9716-45-11-S1.docx]

**Additional file 1 Summary of findings of the previous experiments.**

| **Inocula** | **Early gestation [6]** | | **Mid gestation [7]** | | **Late gestation [9]** | |
| --- | --- | --- | --- | --- | --- | --- |
|  | **Dam Nº** | **Foetus status** | **Dam Nº** | **Foetus status** | **Dam Nº** | **Foetus status** |
| Control | 1 | viable | 1 | viable | 1 | viable |
|  | 2 | viable | 2 | viable | 2 | viable |
|  | 3 | viable | 3 | viable | 3 | viable |
|  | 4 | viable |  |  | 4 | viable |
|  | 5 | viable |  |  |  |  |
|  | 6 | viable |  |  |  |  |
|  | 7 | viable |  |  |  |  |
|  | 8 | viable |  |  |  |  |
| SC | 9 | viable | 4 | viable | 5 | viable |
|  | 10 | viable | 5 | viable | 6 | viable |
|  | 11 | viable | 6 | viable | 7 | viable |
|  | 12 | non-viable | 7 | viable | 8 | viable |
|  | 13 | viable | 8 | viable | 9 | viable |
|  | 14 | non-viable | 9 | viable | 10 | viable |
|  | 15 | viable |  |  | 11 | viable |
|  | 16 | non-viable |  |  | 12 | viable |
|  |  |  |  |  | 13 | viable |
|  |  |  |  |  | 14 | viable |
|  |  |  |  |  | 15 | viable |
| IV | 17 | viable |  |  |  |  |
|  | 18 | viable |  |  |  |  |
|  | 19 | non-viable |  |  |  |  |
|  | 20 | non-viable |  |  |  |  |
|  | 21 | non-viable |  |  |  |  |
|  | 22 | non-viable |  |  |  |  |
|  | 23 | non-viable |  |  |  |  |
|  | 24 | non-viable |  |  |  |  |

Inocula: control (negative control animals inoculated with Vero cells); SC (dams subcutaneously inoculated with 5 × 10^8^ Nc-1 tachyzoites) and IV (dams intravenously inoculated with 5 × 10^8^ Nc-1 tachyzoites). Foetus status: viable (viable foetus when dam was euthanized), non viable (non viable foetus or empty uterus without foetus when dam was euthanized.
