# Supplementary material for: Inflammatory infiltration into placentas of Neospora caninum challenged cattle correlates with clinical outcome of pregnancy
Source: Vet Res. 2014 Jan 31;45(1):11. doi: 10.1186/1297-9716-45-11 (PMC3922085; doi:10.1186/1297-9716-45-11)
Supplement: Additional file 2 — mAb used to identify different immune cell phenotypes in the placentomes. List of the mAb used to overnight incubate the placentomes during the IHC. [file 1297-9716-45-11-S2.docx]

**Additional file 2 mAb used to identify different immune cell phenotypes in the placentas.**

| **Cluster of differentiation** | **Targeted immune cell** | **mAb clone** | **Dilution** |
| --- | --- | --- | --- |
| CD68 | Monocytes/macrophages | EBM11^(1) (a, b, c)^ | 1:100 |
| CD3 | Total T lymphocytes | MM1A^(2) (a, b, c)^ | 1:2000 |
| CD4 | CD4 T lymphocytes | CC30^(3) (c)^ | 1:50 |
|  |  | ILA-12^(4) (a, b)^ | 1:1000 |
| CD8 | CD8 T lymphocytes | CC58^(3) (c)^ | 1:200 |
|  |  | ILA-105^(4) (a, b)^ | 1:200 |
| γδTCR | γδ-T lymphocytes | IL-A29^(2) (a, b, c)^ | 1:4000 |
| CD335 | Natural killer cells | NKp46^(3) (a, b, c)^ | 1:250 |
| CD79_αcy_ | Total B cells | HM57^(1) (a, b, c)^ | 1:100 |

^(1)^ Dako Cytomation, Glostrup, Denmark

^(2)^ VMRD Inc, Washington, USA

^(3)^ AbD Serotec, Oxford, UK

^(4)^ ILRI, International Livestock Research Institute, Nairobi, Kenya

^(a)^ Early, ^(b)^ mid and ^(c)^ late gestation experiments.
