# Supplementary figures and images for: Inflammatory infiltration into placentas of Neospora caninum challenged cattle correlates with clinical outcome of pregnancy
Source: Vet Res. 2014 Jan 31;45(1):11. doi: 10.1186/1297-9716-45-11 (PMC3922085; doi:10.1186/1297-9716-45-11)

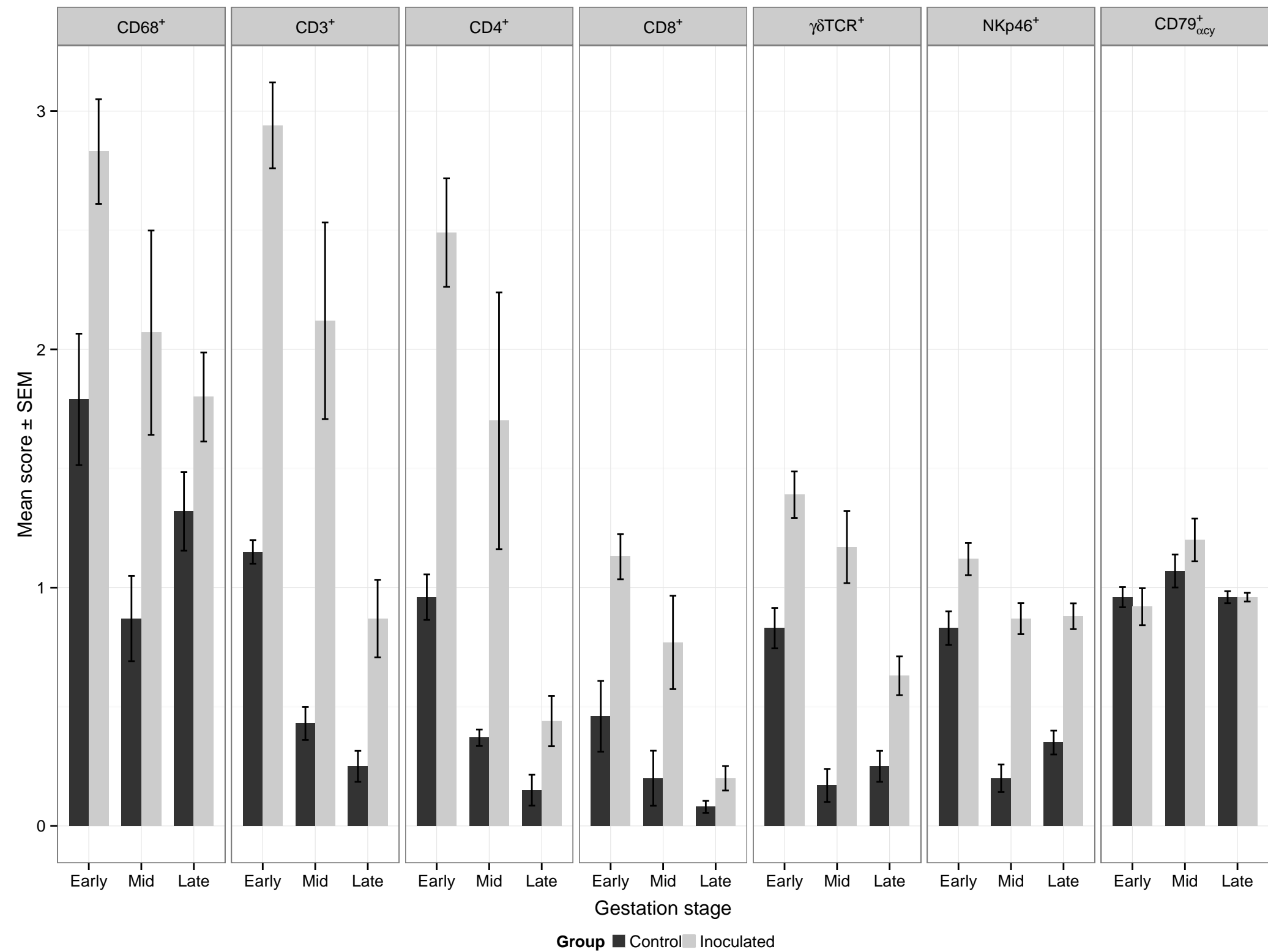

Supplement: Additional file 3 — Infiltration scores in placentomes from negative control and N. caninum-inoculated cows. Mean infiltration score for placentomes collected from negative control (black bars) and N. caninum inoculated (grey bars) cows during the early, mid and late gestation experiments. Error bars indicate standard error of the means. [file 1297-9716-45-11-S3.pdf]
